# Miscellany

**Published:** 1902-03

**Authors:** 


### MISCELLANY.

---

NOTE ON ALBARGIN (Gelatose-silver)—This is an antiseptic, germicide and antigonorrheal remedy, containing 15 per cent of silver, or twice the amount of any other silver proteid. The gelatose molecule is very much smaller than is that of albumin, and this fact allows of a more ready and thorough penetration of the tissues. Because of the greater silver strength, a lesser percentage solution is needed to destroy the various forms of bacteria, 0.1 to 1.0 per cent. being used, according to indications. This fact very materially lessens the cost of treatment also, a not unimportant item.

Albargin is not precipitated from solutions by hydrochloric acid, sodium chlorid solution, nor does it coagulate albumin, thus building an impossible barrier to the antiseptic virtues of silver. This product seems to possess all the requirements of an ideal antiseptic for the treatment of gonorrhea, eye and ear conditions, as well as throat affections, where a remedy of this character is indicated. Messrs. Victor Koechl & Co., of New York, are the agents of this promising remedy.

This firm has also the valerianic acid diethylamid, known by the trade name, valyl. Experiments show valyl to possess all the virtues of valerianic acid and valerian when at its best, unfortunately seldom, as the varying conditions of valerian make its use very uncertain.

Valyl is offered in gelatin capsules, each containing 2 grs. with an equal amount of olive oil, and is recommended in hysteria, fuctional heart disturbances, migrain, hemicrania, neuralgia, and painful menstruation.

---

Laboratory Course in Bacteriology.—Dr. William G. Bissell, bacteriologist to the health department of Buffalo has issued the following circular:

In view of the success attending the laboratory course in bacteriology, which was given to graduates in medicine and dentistry, last winter, in the old laboratory of the Dental Department, of the University of Buffalo, and the fact that a new and finely appointed laboratory is now at my disposal, I have decided to repeat the laboratory course and the first session will be given Monday evening, March 10, 1902, at 8.15 o'clock.

The course will cover six weeks instruction and will include twelve laboratory sessions. Each sesion will be under my personal supervision and the work will cover the more important portion relative to bacterial diagnosis of pus, blood, exudates, sputum, etc., and such parts of bacterial culture media manufacturing, preparation of cultures, staining, etc., as is required in such work. Each session's work will be mapped out in a printed syllabus of original compilation, and which proved such a decided aid to those taking the course last winter. The syllabus has been revised, having received some additions which renders it more serviceable and instructive. Among those who have subscribed to this year's course are Drs. Conrad Diehl, Albert H. Briggs, Herbert Mickle, and others equally well known in the medical profession.

Those desiring to avail themselves of this opportunity to personally carry on practical laboratory diagnosis will please communicate with me on or before March 1, 1902, at whch time the class will be organised.

A fee of fifteen dollars ($15.00) will be charged.

